# Anomalous origin of the left main from the right coronary sinus presenting with sudden cardiac death: utility of mechanical circulatory support

**DOI:** 10.21542/gcsp.2021.24

**Published:** 2021-10-30

**Authors:** Hussam Eddin T. Al Hennawi, Ibrahim Fahsah, Mohammad F. Mathbout

**Affiliations:** 1 Al Faisal University, College of Medicine Riyadh, Saudi Arabia; 2 Norton Healthcare, Department of Cardiology, Louisville, Kentucky, USA; 3 Medical University of South Carolina, Department of Cardiology, Charleston, South Carolina, USA

## Abstract

Anomalies involving the origin of the coronary arteries are extremely rare, with the left main artery coronary artery (LMCA) originating from the right coronary sinus (RCS) one of its rarest forms. Anomalous origin of left main from right coronary sinus poses a high risk of sudden cardiac arrest. In our report, we shed light on the case of a 43-year-old female who suffered a witnessed cardiac arrest due to underlying anomalous origin of the left main artery from right coronary sinus. The patient was initially pronounced dead until return of spontaneous rhythm with concomitant myocardial infarction led to the diagnosis of anomalous coronary artery. This case stresses important points to consider when dealing with the acute management and chronic treatment plan for this subset of high-risk patients. We also consider the utility of mechanical circulatory support in the management of this condition.

## Background

Among different anomalous coronary origins, anomalous origin of the left main coronary artery (LMCA) from the right coronary sinus (RCS) is the most critical, with high risk of imminent sudden cardiac death. Different pathways of origin of coronary arteries have been described, with the most common form being the left circumflex artery (LCX) originating from the RCS. Other forms include both coronary arteries arising from the RCS, the left anterior descending coronary artery from RCS, and a single coronary artery arising from the left sinus of Valsalva. Despite being rare, cases of anomalous coronary arteries can be fatal and most cases are, unfortunately, diagnosed post-mortem. Therefore, a high index of clinical suspicion should be assumed, as early diagnosis could intercept the risk of sudden death and delineate surgical planning with reimplantation of the left main ostium.

## Case presentation

A 43-year-old female was brought in by the emergency medical services (EMS) following a witnessed cardiac arrest. Cardiopulmonary resuscitation (CPR) had immediately been initiated by her husband. En route to the hospital, ACLS protocol was started, and the patient was shocked eight times for VT/VF before she had been pronounced dead in the emergency room (ER).

A few minutes later, her assigned nurse was called to the room by the housekeeper, who-while cleaning the room-had noticed the patient was still spontaneously breathing. The patient’s pulse was faint, with a blood pressure of 83/50. EKG obtained, showing diffuse ST-segment elevation myocardial infarction (STEMI) in the anterior and lateral leads, with reciprocal changes in the inferior leads. STEMI code was activated; the patient was flown to our hospital and was taken immediately to the Cath-lab upon arrival.

**Figure 1.**
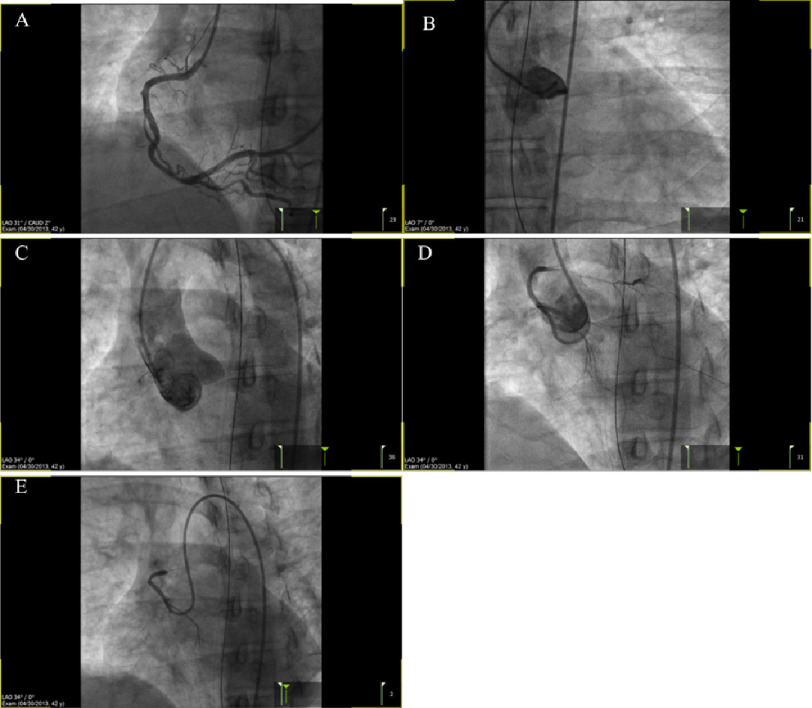
Images obtained during coronary angiography, showing the RCA (A), initial failed attempt to engage the LM (B), non-selective aortic root angiography looking for the LM (C), and selective angiography of the LM (D and E).

Her procedure was complicated by multiple episodes of v-fib arrest, despite which the procedure continued. Diagnostic angiography showed the right coronary artery (RCA) with an initial failed attempt to engage into the left main coronary artery (LMCA). LMCA was successfully outlined using non-selective aortic root angiography followed by selective angiography (Figure 1).

**Figure 2.**
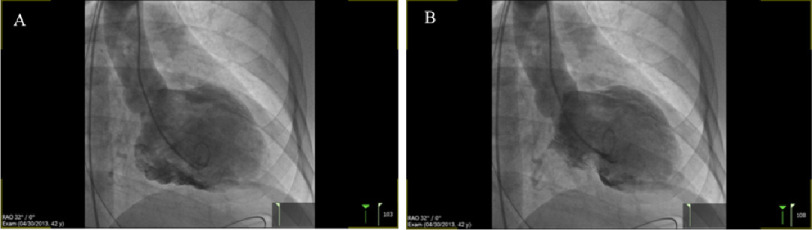
LV angiography showing severely reduced left ventricular systolic function, with the LV in diastole (A) and systole (B).

**Figure 3.**
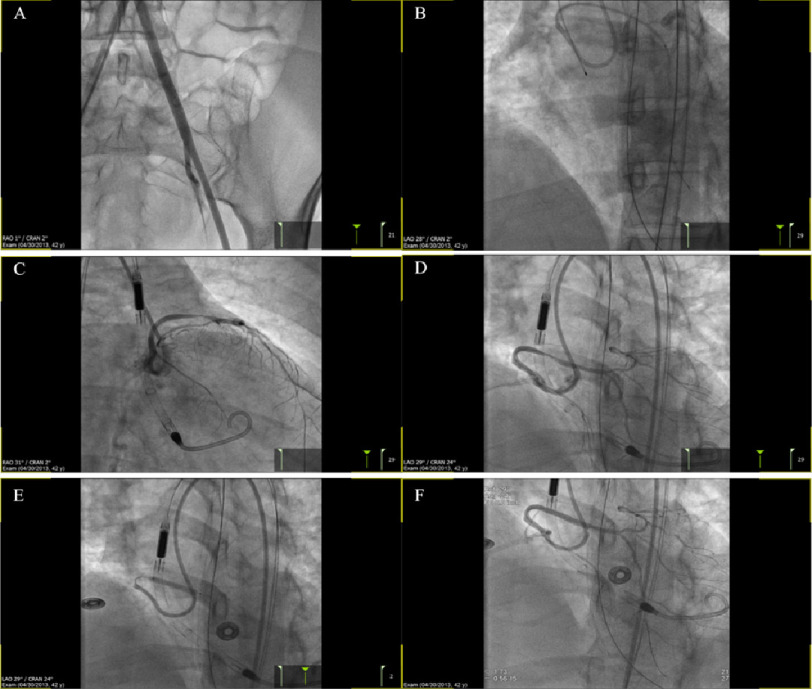
Images show various stages of the intervention, aorto-illiac run off prior to Impella insertion (A), post PTCA (B), following Impella and PTCA (C), post PTCA with a 2.5 balloon (D), stent deployment with Xience 3.0 × 23 mm (E) and following complete revascularization of the vessel (F).

Left ventricular angiography showed severe reduction of left ventricular systolic function (Figure 2). Percutaneous transluminal coronary angioplasty (PTCA) was performed and followed by Impella insertion. Complete revascularization was achieved with a 2.5 balloon and Xience 3.0 × 23 mm stent (Figure 3). Complete revascularization of LMCA was achieved.

The patient was subsequently admitted to the intensive care unit. She suffered from multi-organ failure during her in-patient course. The left ventricular assist device was kept in place for several days for hemodynamic support. The patient recovered and was discharged for rehabilitation therapy.

**Figure 4.**
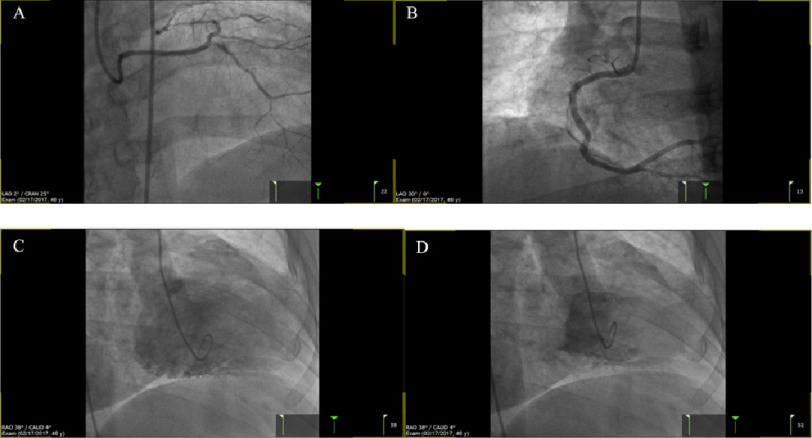
Follow-up left heart catheterization and coronary angiography, four years after the patent’s initial presentation. Images show angiography of the LM (A), RCA (B) and LV angiography in diastole (C) and systole (D).

Four years later, the patient presented with angina, for which she underwent a follow-up left heart catheterization, which showed no obstructive luminal disease (Figure 4).

## Investigations

• Post return of spontaneous rhythm EKG showed diffuse ST-segment elevation in the anterior and lateral leads, with reciprocal changes in the inferior leads.
• Coronary angiography demonstrated initial failed attempt the LM, PTCA with complete revascularization post-Impella CP insertion.
• LV angiography showed severely reduced left ventricular systolic function.
• Follow-up left heart catheterization showed no obstructive luminal disease.

## Discussion

Coronary artery anomalies are considerably rare and challenging to diagnose. Cases are typically discovered in 0.3% of autopsies and in 0.2−0.3% of patients undergoing coronary angiography^1,2^. Common anatomical variants of anomalous coronary artery origins include the circumflex artery stemming from the RCA or the RCS, the RCA arising from the left coronary sinus, or the LMCA originating from the right coronary sinus (present case), which account for 0.05% incidence rate^1^. Anomalous coronary artery origins symptomatology can range from being silent–in which cases happen to be discovered incidentally–to angina, syncope, or fatal sudden cardiac death.

However, treatment guidelines remain controversial, however, and are individualized based on the patient’s risk assessment and symptom burden. Known anomalous coronary artery origin patients presenting with possible ischemic chest pain, syncope linked to ventricular arrhythmias, or previous history of successful sudden cardiac arrest resuscitation should limit exercise, and surgical therapy is typically warranted.

On the contrary, asymptomatic patients must be assessed and risk-stratified. Generally speaking, factors supporting surgical intervention include young patients < 30 years, positive stress testing, high-risk anatomical variants, social factors including the nature of patient’s physical activities, a desire to pursue strenuous sports, and a desire to pursue surgery.

In the current case, clinical presentation in patients with anomalous LMCA from the right coronary sinus differs. Of note, only 20% of patients with underlying anomalous coronary arteries present with symptoms of ischemic heart disease, including angina, dyspnea, or uneventful syncope^3–5^. This raises a great challenge as most cases are first diagnosed at the time of sudden cardiac arrest or post-mortem^3^.

Work up of patients with anomalous coronary arteries is similar to acute coronary syndrome with few points to consider. Electrocardiographic findings in symptomatic patients are nonspecific, ranging from STEMI abnormalities, implying ischemic changes, to ventricular tachycardia or fatal fibrillation^6,7^.

Similar to this case, echocardiographic findings might display left systolic dysfunction, but otherwise, this does not provide any peculiar diagnostic information. Of note, trans-esophageal echocardiography is of limited benefit as this modality does not provide a clear picture of coronary origin and pathway. On the other hand, owing to its fast results and clear depiction of cardiac anatomy, coronary computed tomography angiography (CTA) has shown promising features rendering it the first-line imaging modality in most centers to outline the origin and full pathway of anomalous coronary arteries in patients of interest^6,8^. Moreover, angiographic view of coronary CTA assists in evaluating high-risk anatomical features, including the slit-like origin, acute take-off angle, intramural and elliptical luminal vessel shape and proximal narrowing of anomalous coronary arteries^9,10^.

Mechanical circulatory support (MCS) is increasingly used in settings of cardiogenic shock (CS), anticipated prolonged ischemia/procedure time, and electrical instability in critically ill patients. Mechanical left ventricle (LV) support devices such as Impella CP (Abiomed, Inc., Danvers, Massachusetts) is used to increase stroke volume and/or to alleviate excess end-diastolic left ventricle pressure^11,12^. This unique percutaneous system temporarily reduces LV diastolic pressure, and LV work results in decreased myocardial oxygen demand^13^. This reflects back positively on cardiac output, with resulting improved systemic perfusion and increased coronary flow^14^. The role of Impella CP has been associated with the best clinical profile in cases associated with high-risk PCIs similar to our case, compared to an intra-aortic balloon pump^15^.

Various important clinical implications can be viewed from this report. As with other coronary artery anomalies; an early accurate diagnosis is of paramount importance as this would serve to prevent the risk of sudden death, and aid in surgical planning, with re-implantation of the left main ostium. Most cases are unfortunately diagnosed post-mortem^16,17^. Moreover, outlining the course of the anomalous artery is clinically significant; as this guide surgeons in case of an emergent cardiac surgery or complication at a later course of patients’ survival, for example, during a root dissection in an aortic root replacement or while placing sutures for aortic valve replacement^18^.

## What have we learned?

• Anomalous left main coronary artery (LMCA) originating from the right coronary cusp incidence is low with an approximate incidence of (0.05%).
• Accurate diagnosis is essential, as this would prevent the risk of sudden death and aid in surgical planning, with re-implantation of the left main ostium. Most cases are unfortunately diagnosed post-mortem.
• The role of Impella CP has been associated with the best clinical profile in cases associated with high-risk PCIs, including congenital anomalies of the coronary arteries.
